# Points of Practical Interest in Gynæcology

**Published:** 1901-12

**Authors:** 


**Points of Practical Interest in Gynæcology.** By H. MACNAUGHTON-JONES, M.D., M.Ch., Q.U.I. Pp. ix., 124. London: Baillière, Tindall and Cox. 1901.

This little book consists of reprints of a series of papers in the *Edinburgh Medical Journal*, and is full of practical points presented in a very interesting manner. We quite concur with the author in his powerful advocacy of the means to secure asepsis which he adopts in all his cases, the more especially since in cases of so-called minor gynæcological operations there is a tendency to a dangerous laxity and want of method in preparation shown by many operators. His remarks on curetting are thoroughly sound; we are quite of his opinion that it should be done with the same care as any (so-called) major operation.

Of the six chapters, a most interesting and original one is that on the connection between diseases of the genitalia, neurosis, and insanity. The author brings almost startling evidence to show the large extent to which disease of the pelvic organs is associated with mental disturbance, as shown by records of cases and of *post-mortem* and operation work on the female side of various asylums. This important chapter will well repay careful perusal.

We rather take exception to the use of foreign words, such as "lavabo," in a book of this kind; we fail to see its superiority over "basin-holder" or "washstand." Why not say "wood wool" instead of "Holtzwolle," and save the use of a dictionary?

